# Aerobic Exercise Training Improves Cerebral Blood Flow and Executive Function: A Randomized, Controlled Cross-Over Trial in Sedentary Older Men

**DOI:** 10.3389/fnagi.2019.00333

**Published:** 2019-12-04

**Authors:** Jordi P. D. Kleinloog, Ronald P. Mensink, Dimo Ivanov, Jos J. Adam, Kamil Uludağ, Peter J. Joris

**Affiliations:** ^1^Department of Nutrition and Movement Sciences, NUTRIM School of Nutrition and Translational Research in Metabolism, Maastricht University, Maastricht, Netherlands; ^2^Department of Cognitive Neuroscience, Faculty of Psychology and Neuroscience, Maastricht University, Maastricht, Netherlands; ^3^Department of Biomedical Engineering, N Center, Sungkyunkwan University, Suwon, South Korea; ^4^Techna Institute & Koerner Scientist in MR Imaging, University Health Network, Toronto, ON, Canada

**Keywords:** aging, arterial spin labeling, cerebral blood flow, cognition, exercise, glucose metabolism

## Abstract

**Background:**

Physical activity may attenuate age-related cognitive decline by improving cerebrovascular function. The aim of this study was therefore to investigate effects of aerobic exercise training on cerebral blood flow (CBF), which is a sensitive physiological marker of cerebrovascular function, in sedentary older men.

**Methods:**

Seventeen apparently healthy men, aged 60–70 years and with a BMI between 25 and 35 kg/m^2^, were included in a randomized, controlled cross-over trial. Study participants were randomly allocated to a fully-supervised, progressive, aerobic exercise training or no-exercise control period for 8 weeks, separated by a 12-week wash-out period. Measurements at the end of each period included aerobic fitness evaluated using peak oxygen consumption during incremental exercise (VO_2__peak_), CBF measured with pseudo-continuous arterial spin labeling magnetic resonance imaging, and post-load glucose responses determined using an oral glucose tolerance test (OGTT). Furthermore, cognitive performance was assessed in the domains of executive function, memory, and psychomotor speed.

**Results:**

VO_2__peak_ significantly increased following aerobic exercise training compared to no-exercise control by 262 ± 236 mL (*P* < 0.001). CBF was increased by 27% bilaterally in the frontal lobe, particularly the subcallosal and anterior cingulate gyrus (cluster volume: 1008 mm^3^; *P* < 0.05), while CBF was reduced by 19% in the right medial temporal lobe, mainly temporal fusiform gyrus (cluster volume: 408 mm^3^; *P* < 0.05). Mean post-load glucose concentrations determined using an OGTT decreased by 0.33 ± 0.63 mmol/L (*P* = 0.049). Furthermore, executive function improved as the latency of response was reduced by 5% (*P* = 0.034), but no changes were observed in memory or psychomotor speed.

**Conclusion:**

Aerobic exercise training improves regional CBF in sedentary older men. These changes in CBF may underlie exercise-induced beneficial effects on executive function, which could be partly mediated by improvements in glucose metabolism. This clinical trial is registered on ClinicalTrials.gov as NCT03272061.

## Introduction

People over the age of 60 years represent 13% of the global population and this number is expected to increase at a rate of approximately 3% per year (Department of Economic and Social Affairs Population Division, 2017). Aging is associated with decreased cognitive performance, which is related to decreased cerebrovascular function (Koekkoek et al., 2015; Leeuwis et al., 2018). As impaired cerebrovascular function may precede the decrease in cognitive performance (Jansen et al., 2016; Wolters et al., 2017; Kleinloog et al., 2018), improving cerebrovascular function is an important target to delay cognitive impairment (Gorelick et al., 2011; Joris et al., 2018). In this respect, interventions to improve cerebral blood flow (CBF), a physiological marker of cerebrovascular function (Brown et al., 2007; Liu and Brown, 2007), are of major interest.

A healthy lifestyle, consisting of a healthy diet combined with increased physical activity has been proposed to protect against cognitive impairment by improving CBF (Gorelick et al., 2011). In fact, CBF was improved following a healthy lifestyle intervention and associated with higher cognitive performance in overweight or obese participants aged between 45 and 76 years (Espeland et al., 2018). Furthermore, cross-sectional studies have observed that lower aerobic fitness in sedentary older individuals was associated with a reduced CBF and decreased cognitive performance (Tarumi and Zhang, 2017). A recent meta-analysis of randomized controlled trials involving adults over the age of 50 years showed that aerobic exercise training improved cognitive performance (Northey et al., 2017). This improvement may relate to changes in CBF, since some studies suggest that CBF in the anterior cingulate and hippocampal brain regions increased following aerobic exercise training in sedentary older individuals (Burdette et al., 2010; Chapman et al., 2013; Maass et al., 2015). These exercise-induced changes in hippocampal CBF were positively related to changes in cognitive memory tasks (Chapman et al., 2013; Maass et al., 2015). Therefore, we concluded in our recent review that increases in CBF may contribute to the beneficial effects of increased physical activity levels on cognitive performance (Joris et al., 2018).

However, well-controlled trials investigating the effect of physical activity on CBF are scarce. In some studies, aerobic fitness was measured using a proxy measure which did not improve (Burdette et al., 2010) or was not measured at all (Consortium TtB, 2017), or improvements in aerobic fitness did not sustain (Chapman et al., 2013). Additionally, two studies primarily focused on the hippocampal region, potentially missing changes outside this region (Burdette et al., 2010; Maass et al., 2016). The objective of the current randomized, cross-over trial was therefore to investigate effects of a well-controlled 8-week aerobic exercise training period on CBF and cognitive performance. The study was performed in sedentary overweight or slightly obese older men, because this cohort has been shown to have a reduced CBF and cognitive performance at baseline (Willeumier et al., 2011; Birdsill et al., 2013; Tarumi and Zhang, 2017; Espeland et al., 2018).

## Materials and Methods

### Study Participants

Apparently healthy overweight or slightly obese men were recruited via posters in university and hospital buildings or advertisement in local newspapers. Additionally, participants who had participated in previous studies at Maastricht University were approached if they had given written consent to contact them again for future studies. Volunteers were invited for a screening visit if they met the following inclusion criteria: aged between 60 and 70 years old, body mass index (BMI) between 25 and 35 kg/m^2^; stable body weight (weight gain or loss <3 kg in the past 3 months); non-smoker; no drug or alcohol abuse; no use of dietary supplements known to interfere with the main study outcomes; no diabetes; no use of medication known to affect blood pressure, lipid or glucose metabolism; no severe medical conditions that might interfere with the study (e.g., active cardiovascular disease); and no participation in another biomedical study within 1 month prior to the screening visit.

During screening, sedentary behavior was assessed by means of the international physical activity questionnaire (IPAQ) long version (Craig et al., 2003); an MRI screening list was completed; office blood pressure was measured; a 12-lead electrocardiogram (ECG) was performed; and a fasting blood sample was drawn. Based on the screening results, participants were checked against the following inclusion criteria: classified as low physically active according to the guidelines for IPAQ data processing (IPAQ Research Committee, 2005); no contra-indications for MRI imaging (e.g., any metallic implants or claustrophobia); systolic (SBP) <160 mmHg and diastolic blood pressure (DBP) <100 mmHg, no ECG abnormalities as assessed by a cardiologist; fasting plasma glucose <7.0 mmol/L, fasting serum total cholesterol <8.0 mmol/L, and fasting serum triacylglycerol <4.5 mmol/L. All participants provided written informed consent before screening. The study was conducted according to the guidelines laid down in the Declaration of Helsinki, approved by the Medical Ethics Committee of Maastricht University Medical Center (METC173025), and registered on September 07, 2017 at ClinicalTrials.gov (NCT03272061).

### Study Design

The study had a randomized, controlled cross-over design with an 8-week intervention period and an 8-week control period, separated by a 12-week wash-out period. Participants were allocated to start either in the intervention or control period based on a computer-generated randomization scheme. Participants and investigators were unaware of the allocation prior to inclusion but could not be blinded during the intervention and measurements. However, images and blood samples were blinded prior to analysis. During the intervention period, participants followed a fully-supervised, personalized and progressive aerobic-based exercise program on a cycling ergometer for 50 min three times a week. To personalize the program, maximal exercise capacity (VO_2__max_) and maximal workload (P_max_) were reassessed every 2 weeks and training loads were adjusted accordingly. The program consisted of 10 min warm-up at 45% P_max_, 30 min at 70% P_max_ and 10 min cool-down at 45% P_max_. We did not offer a program during the control and wash-out periods, and participants were requested to maintain habitual physical activity levels during the entire trial. Body weight was measured every 2 weeks in the intervention period and every 4 weeks in the control period. Participants were requested to maintain their habitual diet and use of alcoholic beverages throughout the total trial, which was checked using a food frequency questionnaire.

Measurements were performed at the start of the control and intervention periods (baseline; BL), after 4 weeks (WK4) and during two follow-up days (FU) at the end of each period. The first follow-up visit (FU-1) was performed 43 (range: 19–72) hours after the last training. The second follow-up visit (FU-2) was performed 117 (range: 70–118) hours after the maximal exercise test performed during FU-1. A schematic overview of the study design is shown in Supplementary Figure S1. On the days preceding measurements, participants were requested to have a regular meal and to refrain from alcohol. Participants arrived after an overnight fast (no food or drink after 08:00 PM, except for water) at the Scannexus research facilities in Maastricht (FU-1) or the metabolic research unit Maastricht (MRUM) (FU-2). Men were asked to come by public transport or by car to standardize measurements as much as possible. All measurements were performed in temperature-controlled rooms at 22°C.

### Maximal Exercise Test

Peak oxygen consumption (VO_2__peak_) was assessed every 2 weeks during the intervention period, and three times during the control period at BL, WK4, and FU-1. Heart rate was monitored simultaneously using a chest strap (Sport-tester Polar H10, Kempele, Finland). Thirty minutes before each maximal exercise test, the participants received a small carbohydrate rich meal including a banana and white bread with strawberry jam to optimize performance. The VO_2__peak_ test included an incremental step-wise protocol on a calibrated bicycle ergometer (Lode Excalibur Sport 1000W/1.5V, Groningen, Netherlands), while oxygen consumption (VO_2_) and carbon dioxide production (VCO_2_) were measured continuously (Omnical, Maastricht University, Netherlands).

The VO_2__peak_ test started with a 5-min warm-up at a load of 70W. Workload was increased by 50W every 2.5 min. When anaerobic threshold was observed, workload was increased by 25W every 2.5 min until exhaustion. The anaerobic threshold was determined when the respiratory exchange ratio (RER) was between 0.95 and 1.00. Participants had to reach an RER of at least 1.0 to fulfill the criteria of maximal exertion. P_max_ was calculated as the workload completed (P_completed_) plus time (t) in the last step divided by 150 and multiplied with the load increment of the final stage (ΔW): ${\text{P}}_{\text{max}}\,={\text{P}}_{\text{completed}}+\frac{t}{150}\times \Delta \,W$. Pedal frequency had to be at least 80 RPM. The exercise test was ceased when the pedal frequency remained below 60 RPM for 10 s. VO_2__peak_ was determined as the maximal oxygen consumption for 5 s.

### MRI Acquisition

Scans were performed in the morning of FU-1 on a 3T MAGNETOM Prisma Fit MRI-system using a 64-channel head-neck coil (Siemens Healthcare, Erlangen, Germany). Participants were placed in the scanner with their head-first in the supine position. The eye centers were taken as a reference for the magnet isocenter position, which was at the level of the pons to minimize B_0_ offsets in the labeling region. The labeling plane was positioned perpendicular to the carotid and vertebral arteries, based on an acquired angiogram (TR 21 ms, TE 7.3 ms, voxel volume 0.9 mm × 0.9 mm × 5.0 mm, 8 degrees flip angle, 26 sagittal slices, duration: 2 min).

**FIGURE 1:**
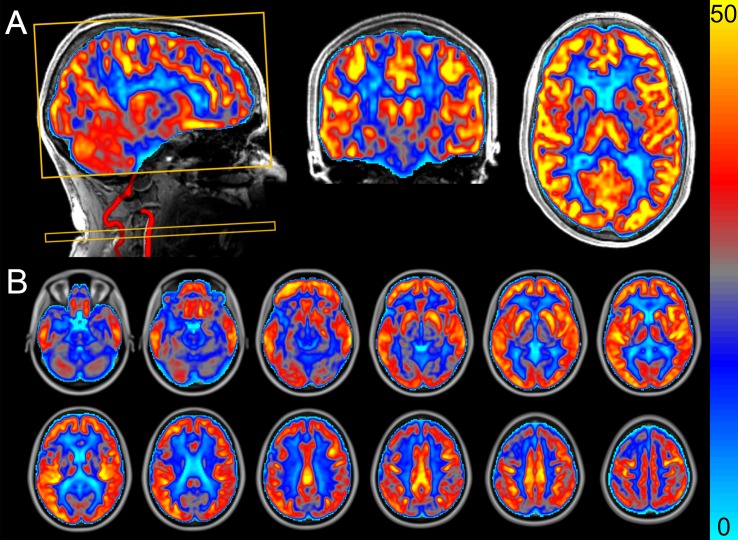
Perfusion-weighted image acquired using pseudo-continuous arterial spin labeling that we generated at the Scannexus research facilities in Maastricht. The images show the cerebral blood flow (CBF) in mL/100 g tissue/min (scale shown by color bar). **(A)** Sagittal slice including angiogram from vertebral and carotid artery, coronal slice, and axial slice. The yellow rectangular boxes represent the imaging box and labeling plane perpendicular to the arteries. **(B)** Mean CBF map from all participants (*n* = 14) after the control period. Data from a randomized, controlled crossover study with sedentary older men.

Perfusion-weighted images were acquired after an acclimatization period of at least 20 min. During acquisition, participants were asked to look at the center of a displayed black cross to standardize measurements as much as possible and to reduce involuntary movements. Images were acquired using pseudo-continuous arterial spin labeling (PCASL) with background-suppressed segmented three-dimensional (3D) gradient and spin echo (GRASE) readouts. The sequence parameters were: TR 4000 ms, TE 13.6 ms, GRAPPA 2, labeling duration 1750 ms, post-labeling delay 2000 ms, segmentation factor 6 (the scan time per 3D volume was 24 s), and 10 label-control repetitions. The total acquisition duration, including an equilibrium magnetization scan, was approximately 9 min). Nineteen slices with a voxel resolution of 3.0 mm isotropic were acquired. In order to allow CBF quantification, a M_0_ image without magnetization preparation and with a TR of 20 s was acquired as well. Figure 1A shows a typical perfusion-weighted image that we generated at the Scannexus research facilities in Maastricht.

One high-resolution anatomical 3D magnetization-prepared rapid acquisition with gradient echo (MPRAGE) scan was performed (TR 2400 ms, TE 2.18 ms, TI 1040 ms, 1.0 mm isotropic resolution, 8 degrees flip angle and 160 sagittal slices, duration: 6 min). The field of view across the various sequences was kept constant for accurate registration and anatomical localization.

### MRI Processing

Volbrain was used to perform brain extraction, along with tissue segmentation for the anatomical MPRAGE image (Manjón and Coupé, 2016). Motion correction was automatically performed by Siemens’ scanner software. FSL software (Version 6.0) was used to estimate quantitative CBF maps from the ASL data^1^. Pairwise subtraction of label and control images was performed on the PCASL data to generate perfusion-weighted images. Perfusion-weighted images were quantified using the BASIL tool (version 4.0.4) (Chappell et al., 2009). following the recommendations of the ASL White Paper (Alsop et al., 2015) The M_0_ image was used for voxel-wise calibration to quantify the perfusion-weighted images. The labeling efficiency was calculated based on the efficiency of four background suppression pulses (0.93^4^), which resulted in a cumulative labeling efficiency of 0.64. The T_1_ of blood depends on the blood hemoglobin concentrations (ctHb) and was estimated using the following equation: 1000/T_1__a_ (ms) = 0.016 × ctHb (g/dL) + 0.317 (Li et al., 2017). The used T_1_ of gray matter was 1330 ms, while the bolus arrival time was set at 1300 ms.

The calibrated ASL images containing absolute CBF values in mL/100 g tissue/min were co-registered using Boundary-Based Registration to the brain-extracted MPRAGE image using the FLIRT routine (Jenkinson et al., 2002). The gray matter partial volume estimates image was thresholded at 0.6 and binarized to create a mask. The mean gray matter CBF was calculated in the anatomical space by taking the mean CBF over the gray matter mask.

The CBF images in anatomical space were registered to MNI (2 mm) space using a non-linear algorithm (FNIRT) and were used for voxel-wise statistical group comparisons. Voxel-wise analyses was performed to detect significantly changed clusters between the intervention and control periods over the whole brain without prior region of interest (Astrakas and Argyropoulou, 2010). These absolute CBF images in MNI space were spatially smoothed with a Gaussian kernel of 1 mm to account for small regional differences, which still existed across participants. Thereafter, a repeated measures mixed-effects analysis using a general linear model with a single-group paired difference (FLAME stage 1 and 2) was used to generate an image containing *Z*-scores for each voxel (Woolrich et al., 2004). Cluster information was extracted after correcting for family-wise error using a Z-threshold of 2.3 (*P* < 0.05) and smoothness estimates, which were computed using the Gaussian Random Field model based on the residual error in each participant. The average probability of the location of the significant clusters was determined using the Atlasquery function in combination with the image of the cluster and the Harvard-Oxford (sub)cortical structural atlas.

### Blood Sampling and Oral Glucose Tolerance Test

During both periods, fasting blood samples were taken at the same time in the morning from a forearm vein by venipuncture at BL, at WK4, and at FU-1. During FU-2, a 7-point oral glucose tolerance test (OGTT) was performed as a measure of peripheral glucose metabolism. For this, blood samples were taken from an intravenous catheter at baseline (*t* = 0 min), and 15, 30, 45, 60, 90, and 120 min following ingestion of 75 g glucose (Novolab, Geraardsbergen, Belgium). After blood sampling, NaF-containing vacutainer tubes (Becton, Dickson and Company, Franklin Lanes, NY, United States) were immediately placed on ice and centrifuged within 30 min at 1300 × *g* for 15 min at 4°C to obtain plasma samples. Blood drawn in vacutainer SST^TM^ II Advance tubes (Becton, Dickson and Company, Franklin Lanes, NY, United States) were first allowed to clot for at least 30 min at 21°C. These tubes were centrifuged at 1300 × *g* for 15 min at 21°C to obtain serum samples. Plasma and serum samples were immediately portioned into aliquots, frozen in liquid nitrogen, and stored at −80°C until analysis at the end of the study.

Plasma obtained from NaF tubes was used to determine glucose concentrations (Horiba ABX, Montpellier, France). Fasting serum samples were analyzed for insulin (RIA, Millipore, Billerica, MA, United States). The net incremental area under the curve (net iAUC) was calculated as described (Brouns et al., 2005). The homeostatic model assessment index was calculated as a measure of insulin resistance (HOMA-IR) (Matthews et al., 1985).

### Cognitive Performance

Cognitive performance was assessed in silent chambers at FU-2 using the Cambridge neuropsychological test automated battery (CANTAB)^2^. These validated, computerized assessments (Robbins et al., 1994, 1998; Louis et al., 1999) have been extensively described^2^ before and measures performance in three cognitive domains: executive function, memory, and psychomotor speed. Based on the literature (Colcombe and Kramer, 2003; Angevaren et al., 2008; Guiney and Machado, 2013; Voss et al., 2013), the main hypothesis for the cognition parameters was that the reaction latency would improve following aerobic exercise training.

Executive function was assessed with the multitasking test (MTT) and spatial span (SSP). Focus was on four variables for the MTT: (1) incongruency cost (IC) was calculated by subtracting the median latency (ML) of response from the trials that were congruent from the incongruent trials; (2) multitasking cost (MTC) was determined as the difference between the ML of response, in which two rules were used (respond at the side the arrow appears or the direction the arrow points) compared to when only one of the rules was used; (3) ML of response for all correct trials; (4) The total number of errors (TE). For SSP, the maximal completed span length (SL) was used.

Memory was evaluated with the delayed matching to sample (DMS) and paired associates learning (PAL). The percentage of correctly answered trials for all delays (CAD) was used for DMS, while the first attempt memory score (FAMS) and TE were used for PAL.

Measurements of psychomotor speed included the motor screening task (MOT) and reaction time (RTI). The mean latency (LM) from target stimulus appearance to button press) outcome variable was used for MOT. For RTI the variables reaction time (RT) from target stimulus appearance to release of response button) and movement time (MT) from release of response button to selection of target stimulus) were used.

### Statistical Analysis

Results are shown as mean ± standard deviation (SD), unless otherwise indicated. Before the start of the study, it was calculated that 15 participants were needed to reach a power of 80% to detect a true difference of 15% in CBF, which was the primary outcome parameter. For these calculations, a two-sided alpha of 0.05 and a within-subject variability of 19% were used (Gevers et al., 2011). CBF changes of 15% may be expected and are also clinically relevant (Alsop et al., 2000; Alexopoulos et al., 2012; Birdsill et al., 2013).

Intervention effects were examined using analysis of variances (ANOVA) with participant, treatment and period as fixed factors. Linear mixed models were performed to test for differences between treatments over time, using the change from baseline as dependent variable. Time, treatment, period and time ^∗^ treatment interaction were used as fixed factors. If the interaction term was not statistically significant, it was omitted from the model. Bonferroni correction was used to correct for multiple comparisons. Statistical analyses were performed using SPSS (IBM Corp., IBM SPSS Statistics, V23, Armonk, NY, United States). Differences were considered statistically significant at *P* < 0.05 using two-tailed tests.

## Results

### Study Participants

**FIGURE 2:**
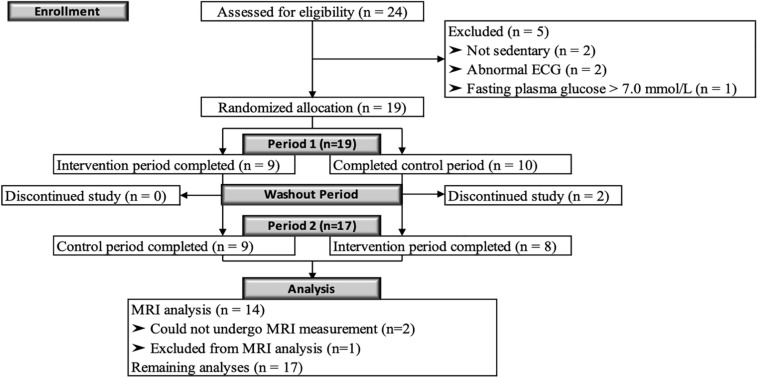
CONSORT flow diagram. Diagram of the progress through the phases of this randomized, controlled crossover study with sedentary overweight or slightly obese older men.

A CONSORT flow diagram of participants throughout the study is shown in Figure 2. In total 24 men were screened for eligibility. Five participants were excluded because they were not sedentary (two men), had an abnormal ECG (two men) or a fasting plasma glucose concentration above 7.0 mmol/L (one man). Thus, nineteen men were eligible and started the study. Two participants who started in the no-exercise control period dropped-out during the wash-out period for personal reasons and seventeen participants successfully completed the study. Two participants did not undergo the MRI measurements: one man became unexpectedly claustrophobic and another man due to remains of a metal screw in his skull following surgery which did not become apparent during the screening visit. Additionally, MRI data from one participant were excluded, because the magnetic field disturbance due to his tooth implant reduced the labeling efficiency of arterial blood. In total, fourteen participants were included in the final MRI analysis, while all seventeen participants were included in all other analyses. Baseline characteristics are shown in Table 1. Participants who completed the study had a mean age of 67 ± 2 years and a mean BMI of 30.3 ± 2.8 kg/m^2^. Body weight remained stable at the follow-up measurements between both periods (0.9 ± 3.0 kg). The median attendance of the scheduled training sessions was 100% (range: 92 – 100%).

**TABLE 1:** Baseline characteristics of sedentary older men who completed the study (*n* = 17).

**Participant characteristics**	
Age (y)	67 ± 2
BMI (kg/m^2^)	30.3 ± 2.8
Total cholesterol (mmol/L)	5.28 ± 1.10
TAG (mmol/L)	1.39 ± 0.49
Glucose (mmol/L)	5.80 ± 0.36
Systolic blood pressure (mmHg)	138 ± 13
Diastolic blood pressure (mmHg)	88 ± 6

*Data are shown as mean ± SD. BMI, body mass index; TAG, triacylglycerol.*

### Maximal Exercise Test

**FIGURE 3:**
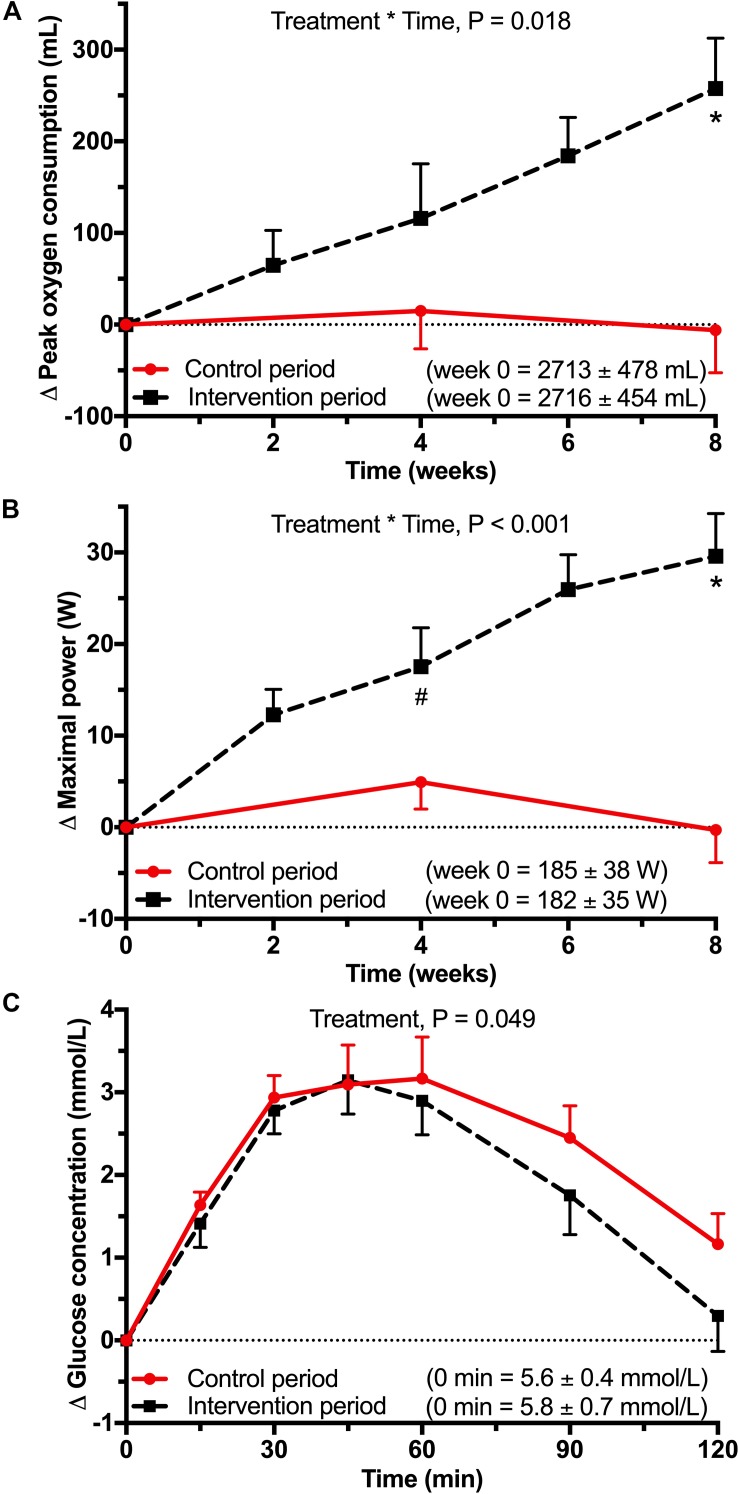
Data from a randomized, controlled crossover study with sedentary overweight or slightly obese older men (*n* = 17). Data were analyzed using linear mixed models on the difference between each timepoint with baseline. **(A)** Mean (±SEM) difference in peak oxygen consumption (VO_2__peak_) and **(B)** maximal power (P_max_) difference during the maximal exercise. Maximal exercise tests were performed every 2 weeks during the intervention period. During the control period, maximal exercise tests were performed at baseline, after 4 weeks and after 8 weeks. Baseline values were not significantly different. There was a significant treatment ^∗^ time interaction for VO_2__peak_ (*P* = 0.018) and P_max_ (*P* < 0.001). After Bonferroni correction there was a significant difference between control and intervention period at 4 weeks (^#^*P* = 0.006) and at 8 weeks for VO_2__peak_ and P_max_ (^∗^*P* < 0.001). **(C)** Mean (±SEM) difference in glucose concentrations during a 7-point oral glucose tolerance test (OGTT) test. There was a significant treatment effect for glucose concentration (*P* = 0.049).

All men reached a RER of at least 1.0 during all maximal exercise tests (1.12 ± 0.05), suggesting maximal exertion was reached. As expected, physical exercise training significantly increased aerobic fitness, as indicated by the significant time ^∗^ treatment interaction for the VO_2__peak_ (*P* = 0.018), using linear mixed models. Pairwise comparisons showed that the VO_2__peak_ tended to increase by 99 ± 236 mL (*P* = 0.088) during the intervention period at week 4 and was significantly increased by 262 ± 236 mL (*P* < 0.001) at week 8 (Figure 3A). Comparable results were observed for P_max_ (time ^∗^ treatment interaction: *P* < 0.001), which increased during the intervention at week 4 by 12 ± 18 W (*P* = 0.006) and at week 8 by 30 ± 18 W (*P* < 0.001; Figure 3B).

### Cerebral Blood Flow

**FIGURE 4:**
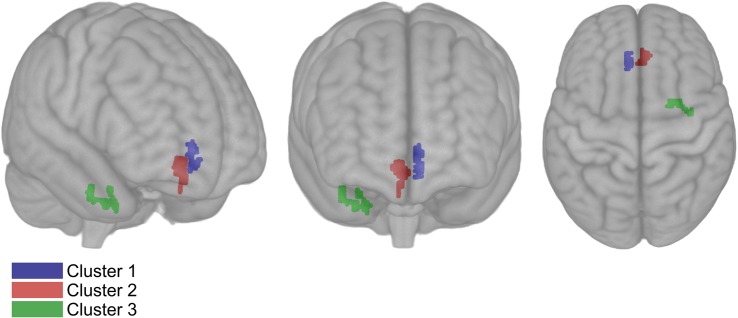
Results of voxel-wise comparisons including all acquired cerebral blood flow (CBF) data in three dimensional MNI template from a randomized, controlled crossover study with sedentary overweight, or slightly obese older men (*n* = 14). CBF increased bilaterally after the intervention compared to the control period *P* < 0.05 (Family-wise error corrected). The CBF of cluster 1 and 2 increased after the intervention period compared to the control with 6.39 mL/100 g tissue/min (volume: 392 mm^3^) and 6.95 mL/100 g tissue/min (volume: 616 mm^3^), respectively. The CBF of cluster 3 decreased with 4.4 mL/100 g tissue/min (volume: 408 mm^3^).

The mean CBF map from all participants after the control period is shown in Figure 1B. CBF differed between intervention and control periods in three clusters with a volume of 392 mm^3^ (cluster 1), 616 mm^3^ (cluster 2) and 408 mm^3^ (cluster 3) (Figure 4 and Table 2). CBF was increased by 28% (6.4 ± 5.0 mL/100 g tissue/min; *P* = 0.040) in cluster 1 and by 26% (7.0 ± 4.8 mL/100 g tissue/min; *P* = 0.001) in cluster 2 after the intervention period. In contrast, CBF was decreased by 19% (−4.4 ± 1.9 mL/100 g tissue/min; *P* = 0.031) in cluster 3. The average probabilities for the locations of cluster 1 were 25% in the subcallosal cortex, 11% in the anterior cingulate gyrus, 8% in the paracingulate gyrus, and 3% in the frontal medial cortex. For cluster 2, which was located contralateral to cluster 1, the average probabilities were 24% in the subcallosal cortex, 23% in the frontal medial cortex, 15% in the paracingulate gyrus, and 12% in the anterior cingulate gyrus. For cluster 3, these probabilities were 35% in the temporal fusiform cortex and 25% in the parahippocampal gyrus.

**TABLE 2:** Mean ± SD cerebral blood flow difference between intervention and control period in a randomized, controlled crossover study with sedentary older men (*n* = 14).

	**Intervention period** **(mL/100 g/min)**	**Control period** **(mL/100 g/min)**	**Mean difference** **(mL/100 g/min)**	***P***
Gray matter CBF	27.6 ± 9.4	28.4 ± 7.9	−0.6 ± 3.6	0.533
Global CBF	23.5 ± 7.8	24.4 ± 6.4	−0.5 ± 3.0	0.523
Left hemi CBF	24.5 ± 8.7	25.4 ± 7.3	−0.5 ± 4.5	0.637
Right hemi CBF	25.7 ± 8.1	26.3 ± 6.7	−0.3 ± 3.9	0.723
Cluster 1 CBF	29.1 ± 12.2	22.7 ± 11.0	6.4 ± 4.8	0.040
Cluster 2 CBF	33.7 ± 17.8	27.0 ± 16.3	7.0 ± 4.9	0.001
Cluster 3 CBF	18.7 ± 5.3	23.1 ± 5.4	−4.4 ± 1.9	0.031

*CBF, cerebral blood flow; hemi, hemisphere.*

We did not observe differences between the intervention and control periods (Table 2) in global CBF (−0.5 ± 3.0 mL/100 g tissue/min; *P* = 0.523), gray matter CBF (−0.6 ± 3.6 mL/100 g tissue/min; *P* = 0.533), CBF in the left hemisphere (−0.5 ± 4.5 mL/100 g tissue/min; *P* = 0.637), and CBF in the right hemisphere (−0.3 ± 3.9 mL/100 g tissue/min; *P* = 0.723).

### Glucose Metabolism

A significant treatment effect was observed for the post-load glucose concentrations measured during the OGTT (*P* = 0.049) after the intervention period (Figure 3C). Pairwise comparisons showed a tendency toward lower glucose concentrations at 120 min (−0.86 ± 1.91 mmol/L; *P* = 0.083). Also, the net iAUC tended to decrease (−45 ± 96 mmol/L^∗^2 h; *P* = 0.072).

Linear mixed models showed no time ^∗^ treatment interactions for fasting glucose (*P* = 0.131) and insulin (*P* = 0.949) concentrations and the HOMA-IR (*P* = 0.772). There were also no significant treatment effects when this interaction term was omitted from the model (glucose: *P* = 0.146; insulin: *P* = 0.390; HOMA-IR: *P* = 0.423).

### Cognitive Performance

Performance on the executive function MTT improved as indicated by a significant decrease in ML (−37 ± 65 ms; *P* = 0.034), while the number of total errors remained the same (0 ± 6; *P* = 0.770). The other MTT variables did not change. In addition, no changes were observed for the executive function test SSP, the memory tests DMS and PAL, and the psychomotor speed tests MOT and RTI (Table 3).

**TABLE 3:** Mean ± SD between intervention and control period of cognitive outcomes in a randomized, controlled crossover study with sedentary older men (*n* = 17).

	**Intervention period**	**Control period**	**Mean difference**	***P***
MOT ML (ms)	846 ± 167	847 ± 177	−1 ± 157	0.980
RTI MT (ms)	281 ± 67	289 ± 56	−8 ± 56	0.574
RTI RT (ms)	399 ± 32	405 ± 45	−5 ± 33	0.510
MTT IC (ms)	117 ± 53	112 ± 48	4 ± 45	0.724
MTT MTC (ms)	281 ± 107	264 ± 109	17 ± 161	0.675
MTT RL (ms)	753 ± 87	790 ± 98	−37 ± 65	0.034
MTT IN	7 ± 9	7 ± 10	0 ± 6	0.770
SSP SL	6 ± 1	6 ± 1	0 ± 1	0.414
DMS TC (%)	80 ± 9	83 ± 7	−3 ± 12	0.336
PAL FAMS	11 ± 3	12 ± 2	−1 ± 2	0.282
PAL TE	17 ± 9	16 ± 9	2 ± 8	0.398

*MOT, motor screening task; RTI, reaction time; MTT, multitasking test; SSP, spatial span; DMS, delayed matching to sample; PAL, paired associates leaning; LM, mean: latency; MT, movement time; RT, reaction time; IC, incongruency cost; MTC, multitasking cost; ML, median latency; TE, total errors; SL, forward span length; CAD, total correct; FAMS, first attempt memory score.*

## Discussion

In this well-controlled, randomized trial in sedentary older men, aerobic exercise training affected regional CBF. It increased bilaterally in the subcallosal and anterior cingulate gyrus, which are both located in the frontal lobe. These two regions have been identified as important nodes in the limbic system and are involved in the regulation of executive cognitive functions (Vogt et al., 1992; Hamani et al., 2011). Reduced CBF was observed in one cluster located in the right medial temporal lobe, mainly the temporal fusiform gyrus. In addition, latency of response was reduced for the executive function test and mean post-load glucose concentrations decreased. Recent findings suggest that unfavorable regional CBF alterations underlie reduced cognitive performance in older individuals, which may be mediated by impaired glucose metabolism (Bangen et al., 2018). This underlines the potential clinical relevance of the observed concomitant improvements in regional CBF, glucose metabolism, and executive function following exercise-induced increased fitness.

Cerebral blood flow increased by 27% bilaterally in the frontal lobe in clusters with a total volume of 1008 mm^3^. The location of the cluster was comparable to the cluster identified by Chapman et al. (Chapman et al., 2013). However, they did not quantify the change in CBF, and the volume of the cluster was only 696 mm^3^. Additionally, they did not show sustained increases in aerobic fitness. Therefore, the smaller cluster volume may be explained by the lower effectiveness of the intervention used. Reduced CBF in the frontal lobe is associated with increased age (Zhang et al., 2018), and a 4-year prospective longitudinal study observed that CBF at baseline was associated with cognitive performance at follow-up (De Vis et al., 2018).

Interestingly, CBF decreased by 19% in the right medial temporal lobe and this cluster had a volume of 408 mm^3^. The observed decrease in CBF may attenuate progression of cognitive decline associated with human aging, as shown by a positive correlation of CBF in the temporal lobe with age (Preibisch et al., 2011; Zhang et al., 2018). Hays et al. (2018) have suggested that an increased temporal lobe CBF in a population with declined cognitive performance reflects neurovascular dysregulation. Additionally, increased CBF was observed in the medial temporal lobe early in the development of mild cognitive impairment (Dickerson and Sperling, 2008; Lacalle-Aurioles et al., 2014). In contrast to our findings, Maass et al. (2015) observed a decrease in hippocampal CBF after exercise in older individuals. Pereira et al. (2007) also showed an exercise-induced increase in cerebral blood volume in the dentate gyrus – a subregion of the hippocampus – in young and middle-aged participants. The intervention periods in these studies were 1 to 2 months longer than in our study, and image acquisition techniques were particularly optimized to detect changes in the hippocampus. This may have decreased the sensitivity to detect changes outside their region of interest, while our study may have been less sensitive to detect hippocampal changes due to coarser resolution and related partial volume effects. In addition, it is possible that longer intervention periods are needed to induce CBF changes in hippocampal brain regions. Indeed, Burdette et al. have shown that hippocampal CBF was higher following exercise training (Burdette et al., 2010). However, in this parallel study only post-intervention scans were performed, while the exercise training group consisted of 50% women compared to no women in the control group. Also, CBF was not corrected for hematocrit, which may have resulted in higher CBF values in women (Smith et al., 2019). Therefore, the observed CBF differences may be partly due to gender-mismatch instead of exercise training.

No changes in whole brain or gray matter CBF were observed. Gray matter CBF was based upon individually generated gray-matter masks in native space to ensure optimal overlap between structural gray matter regions and CBF. The mean gray-matter mask volume was comparable between the intervention and control periods within one participant (0.3 ± 3.0%). Gray matter CBF values were comparable with observed blood flow levels in studies that used partial volume correction (Preibisch et al., 2011; Bangen et al., 2018; Hays et al., 2018; Leeuwis et al., 2018) as incorporated in the FSL Basil tool. In fact, the mean gray matter CBF with partial volume correction in our study was 49.8 ± 13.0 ml/100 g tissue/min. However, we did not use this correction, also because the validity of proposed partial volume correction approaches has recently been questioned (Kirk et al., 2019). Decreased gray matter CBF has been observed in sedentary populations (Birdsill et al., 2013; Dai et al., 2017; Tarumi and Zhang, 2017), and was associated with accelerated cognitive decline (Wolters Frank et al., 2017). However, in agreement with our findings, other studies investigating the effect of aerobic exercise training on CBF also did not observe changes at the whole-brain level (Chapman et al., 2013; Maass et al., 2015).

Glucose metabolism improved, as indicated by the decreased post-load glucose concentrations. Besides the well-known effects of aerobic exercise training in (pre-)diabetics (Pan et al., 2018), Ferrara et al. (2006) also observed that exercise training increased glucose disposal in apparently healthy older men. Fasting plasma glucose concentrations did not change, which agrees with the result of a meta-analysis of 105 intervention studies (Boniol et al., 2017). The observed beneficial effects on CBF in specific brain regions may be linked to the improved glucose metabolism. This is supported by the relation between a reduced CBF in the frontal lobe and decreased cognitive performance in type 2 diabetic patients compared to healthy controls (Dai et al., 2017). Nevertheless, a causal relationship between peripheral and brain insulin sensitivity has not yet been established (Kullmann et al., 2015; Arnold et al., 2018).

Several reviews concluded that aerobic exercise training improves executive function (Colcombe and Kramer, 2003; Angevaren et al., 2008; Guiney and Machado, 2013; Voss et al., 2013), which is in line with the current findings. The latency of response decreased when the correct answer was given, while the number of errors remained unchanged, which indicates favorable effects on cognitive performance within the domain of executive function. This decrease in latency may be associated with improved response-inhibition, and was not due to speed-accuracy trade-off (Lo et al., 2015). The favorable effects on cognitive performance are in line with the improvements of CBF in the frontal lobe, which has been identified to be important in executive function (Yuan and Raz, 2014). No changes in cognitive performance in (visuo-spatial) memory or psychomotor speed were observed. Similarly, visuo-spatial memory did not improve following long-term aerobic exercise as shown in a meta-analysis of 21 aerobic exercise training studies, whereas verbal-auditory memory did improve (Roig et al., 2013). Longer intervention periods may be needed to improve visuo-spatial memory, since only one trial with a 12-month physical training intervention showed beneficial effects on visuo-spatial memory (Erickson et al., 2011). Psychomotor speed only increased in studies with combined aerobic exercise- and resistance- or cognitive training (Levin et al., 2017). Multimodal combined training may thus be required to improve performance in psychomotor speed tests.

As expected, aerobic exercise training improved aerobic fitness during maximal exercise. VO_2__peak_ increased significantly by 10% between the intervention and control group after 8 weeks, while P_max_ already increased after 4 weeks. These concomitant increases were expected based on the linear relationship between VO_2__peak_ and P_max_ (Schoffelen et al., 2019). The consistent increase in VO_2__peak_ and P_max_ of our trial emphasizes the effectiveness of the intervention. This may be attributed to a combination of several factors, including (i) the duration, frequency and tightly controlled supervised training sessions; (ii) the individually based progressive training intensity; and (iii) the inclusion of sedentary individuals. Maass et al. also showed an increase of 10% oxygen consumption at ventilatory anaerobic threshold after 12 weeks of 30 min interval training (Maass et al., 2015). In contrast, Chapman et al. only showed a change in VO_2__peak_ at 6 weeks, which did not sustain after 12 weeks of aerobic exercise training (Chapman et al., 2013). Burdette et al. used a proxy-measure (400m walk speed) that did not significantly differ between groups and the duration and intensity of the home-based training sessions was not controlled (Burdette et al., 2010). The Train the Brain Consortium did not measure the effectiveness of the physical exercise training by means of an aerobic fitness outcome (Consortium TtB, 2017). Therefore, it cannot be assessed whether changes in CBF in these studies are due to exercise-induced changes in aerobic fitness.

Our tightly controlled, progressive, aerobic exercise training showed almost perfect attendance by the participants, generating consistent improvements in aerobic fitness across the 8-week intervention period. This trial included only men to reduce gender differences as an extra source of variability, which reduces the external validity. Additionally, although we were properly powered to find changes in our primary outcome, our sample size was too limited to examine into detail relationships between changes in aerobic fitness, CBF, glucose metabolism, and cognitive performance.

## Conclusion

Our results show that aerobic exercise training improves regional CBF in sedentary older men. Also, cognitive performance in the domain of executive function improved, and beneficial effect on peripheral glucose metabolism were observed. Whether the observed exercise-induced changes in CBF underlie the beneficial effects on cognitive performance, and if they are mediated by changes in peripheral and/or brain insulin sensitivity requires further study.

## Data Availability Statement

All data supporting the conclusions of this study are presented in the article.

## Ethics Statement

This study involving human participants was reviewed and approved by the Ethics Committee of Maastricht University Medical Center (METC173025). The participants provided their written informed consent to participate in this study.

## Author Contributions

JK designed and conducted the study, performed the statistical analyses, interpreted the data, developed the analysis pipeline, and wrote the manuscript. RM and PJ designed the study, interpreted the data, had overall responsibility for the study, and wrote the manuscript. DI and KU developed the MRI sequences and analysis pipeline, interpreted the data, and reviewed the manuscript. JA developed the cognitive performance assessment protocol, interpreted the data, and reviewed the manuscript.

## Conflict of Interest

The authors declare that the research was conducted in the absence of any commercial or financial relationships that could be construed as a potential conflict of interest.

## Footnotes

1 http://fsl.fmrib.ox.ac.uk/fsl

2 https://www.cambridgecognition.com/cantab/

## Supplementary Material

FIGURE S1 Schematic overview of study design. Timeline is displayed as weeks. FU-1, follow-up day one; FU-2, follow-up day two.
