# Supplementary figures and images for: Aerobic Exercise Training Improves Cerebral Blood Flow and Executive Function: A Randomized, Controlled Cross-Over Trial in Sedentary Older Men
Source: Front Aging Neurosci. 2019 Dec 4;11:333. doi: 10.3389/fnagi.2019.00333 (PMC6904365; doi:10.3389/fnagi.2019.00333)

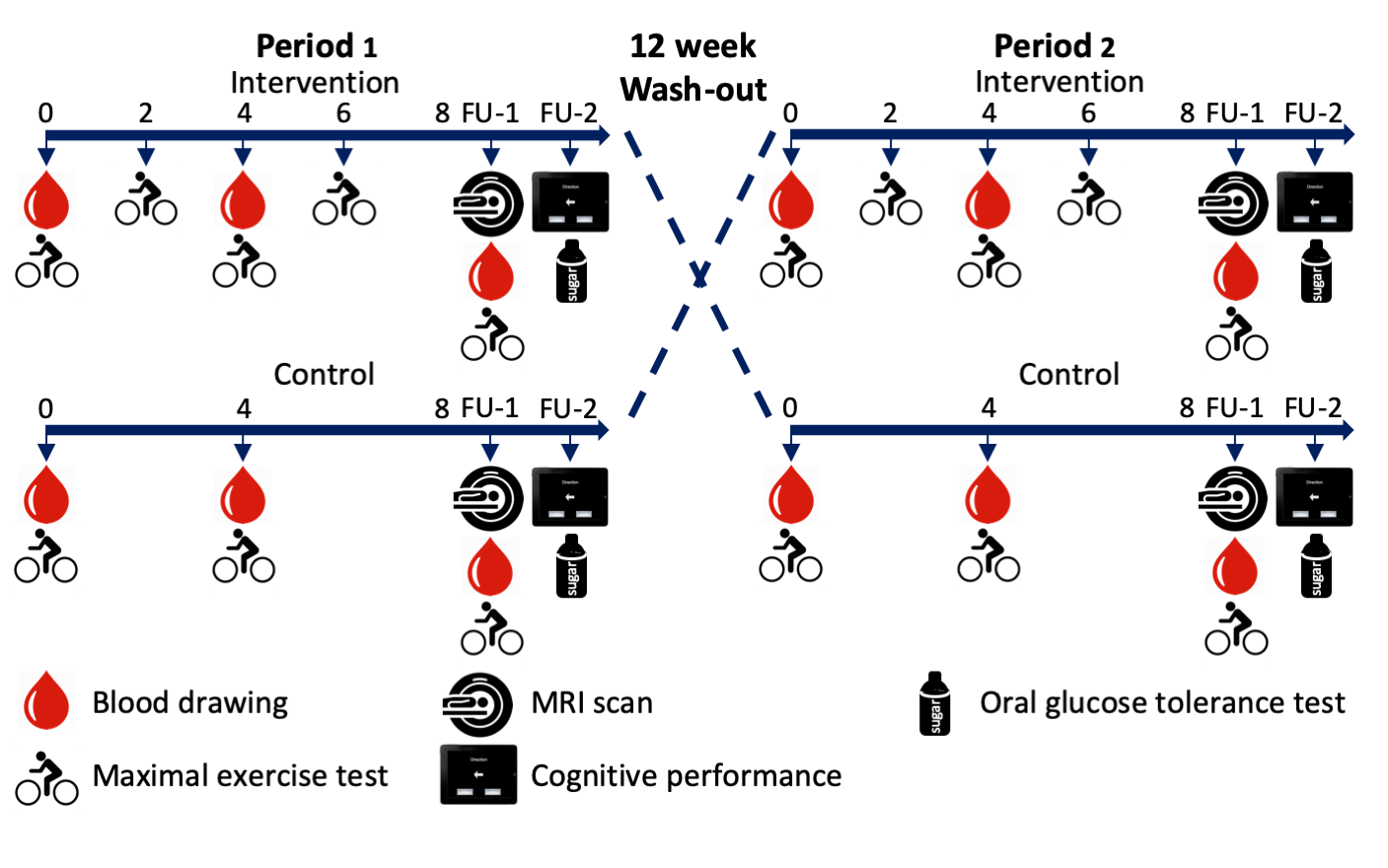

Supplement: FIGURE S1 — Schematic overview of study design. Timeline is displayed as weeks. FU-1, follow-up day one; FU-2, follow-up day two. [file Image_1.TIFF]
